# Mutations in *ap1b1* Cause Mistargeting of the Na^+^/K^+^-ATPase Pump in Sensory Hair Cells

**DOI:** 10.1371/journal.pone.0060866

**Published:** 2013-04-12

**Authors:** Rachel Clemens Grisham, Katie Kindt, Karin Finger-Baier, Bettina Schmid, Teresa Nicolson

**Affiliations:** 1 Oregon Hearing Research Center and Vollum Institute, Howard Hughes Medical Institute, Oregon Health and Science University, Portland, Oregon, United States of America; 2 Max-Planck-Institute for Neurobiology, Dept. Genes - Circuits ­ Behavior, Martinsried, Germany; 3 German Center for Neurodegenerative Diseases (DZNE), Munich, Germany; 4 Adolf-Butenandt-Institute, Biochemistry, Ludwig-Maximilians-University Munich, Munich, Germany; Texas A&M University, United States of America

## Abstract

The hair cells of the inner ear are polarized epithelial cells with a specialized structure at the apical surface, the mechanosensitive hair bundle. Mechanotransduction occurs within the hair bundle, whereas synaptic transmission takes place at the basolateral membrane. The molecular basis of the development and maintenance of the apical and basal compartments in sensory hair cells is poorly understood. Here we describe auditory/vestibular mutants isolated from forward genetic screens in zebrafish with lesions in the *adaptor protein 1 beta subunit 1* (*ap1b1)* gene. Ap1b1 is a subunit of the adaptor complex AP-1, which has been implicated in the targeting of basolateral membrane proteins. In *ap1b1* mutants we observed that although the overall development of the inner ear and lateral-line organ appeared normal, the sensory epithelium showed progressive signs of degeneration. Mechanically-evoked calcium transients were reduced in mutant hair cells, indicating that mechanotransduction was also compromised. To gain insight into the cellular and molecular defects in *ap1b1* mutants, we examined the localization of basolateral membrane proteins in hair cells. We observed that the Na^+^/K^+^-ATPase pump (NKA) was less abundant in the basolateral membrane and was mislocalized to apical bundles in *ap1b1* mutant hair cells. Accordingly, intracellular Na^+^ levels were increased in *ap1b1* mutant hair cells. Our results suggest that Ap1b1 is essential for maintaining integrity and ion homeostasis in hair cells.

## Introduction

Auditory and vestibular hair cells (HCs) are polarized epithelial cells with a unique morphology essential for mechanosensation [1]. Hair bundles at the apical end of HCs are comprised of several rows of actin-filled stereocilia and a single primary cilium called the kinocilium. Upon deflection induced by sound or head movements, hair bundles transduce mechanical stimuli into graded receptor potentials. Within the basolateral compartment, HCs transmit signals to afferent neurons, and in some cases receive signals from efferent neurons [2], [3]. In addition to the transduction and synaptic machinery, a number of channels and transporters are spatially restricted to the apical or basolateral ends of HCs. These proteins are critical for maintaining the electro-chemical gradients necessary for HC function [4], [5], [6], [7], [8], [9]. How the HC orchestrates apical and basolateral trafficking of membrane proteins for its unique requirements has not been explored.

Clathrin-mediated transport requires Adaptor Proteins (APs) that interact with sorting motifs on membrane proteins, providing selectivity in the initial step of transport. Several distinct classes of AP complexes (AP-1, AP-2, AP-3, AP-4) facilitate sorting along various trafficking routes [10]. The Adaptor Protein 1 (AP-1) complex has been shown to mediate trafficking of membrane proteins to the plasma membrane from either the trans-Golgi network (TGN) or recycling endosomes. In polarized epithelial-cells, AP-1 is important for basolateral sorting of cargo proteins [11]. The AP-1 complex is composed of four subunits: γ, β1, σ1 and either µ1A or µ1B [12], [13], [14], [15], [16], [17]. The two different µ-subunits distinguish the AP-1A from the AP-1B complex. As the β1 subunit is common to both AP-1A and AP-1B complexes, for the purposes of this study we will refer to them both simply as the AP-1 complex. The AP-1 complex has been studied primarily in cell culture models, however the role of the AP-1 complex in an intact organism is less well understood. Here we investigate the effect of mutations in the zebrafish β1 subunit of the AP-1 complex (*ap1b1)* on protein sorting and HC function *in vivo*.

We isolated two alleles of *ap1b1* from two independent large-scale ENU mutagenesis screens for auditory and vestibular zebrafish mutants [18] (Tübingen 2000 Screen Consortium). Cloning of the lesions in *ap1b1* revealed two early stop mutations. In the present study, we quantify the vestibular and auditory deficits in *ap1b1* mutants and show that mechanotransduction is compromised in mutant HCs. Though AP-1 has been implicated in the sorting of basolateral membrane proteins, HC synapses appear to be largely intact in *ap1b1* mutants. In contrast, the Na^+^/K^+^-ATPase pump is missorted to the apical surface in HCs. Our results suggest that loss of AP-1-sorting leads to mislocalization of the NKA pump and is likely to account, in part, for the defects associated with *ap1b1* mutant HCs.

## Materials and Methods

### Ethics Statement

This study was performed with the approval of the Oregon Health and Science University Institutional Animal Care and Use Committee and in accordance with NIH guidelines.

### Animal Lines

Zebrafish were kept on a 12 hr light-dark cycle at 28°C. The mutant alleles of *skylab, tm246a* and *t20325* were isolated from two independent ENU mutagenesis screens [18] (Tübingen 2000 Screen Consortium) and maintained in either Tübingen or Top long fin wild-type (WT) backgrounds. The *Tg(myo6b:β-actin-GFP)* and *Tg(myo6b:D3cpv)* cameleon lines have been previously described elsewhere [19].

### Behavioral Assays

Vestibular-induced eye movements were recorded from 5 dpf larvae using techniques described in detail elsewhere [20], [21]. Data was analyzed in Matlab. The auditory escape response was quantified using methods described in [22]. Statistical analysis was performed using Prism 5 (GraphPad).

### Cloning and Molecular Biology

The *skylab* critical interval was determined by crossing WT WIK fish with Tübingen fish heterozygous for the mutation. Genetic mapping with F2 mutant larvae was performed through PCR amplification of SSLP markers.

Genes within the critical interval were analyzed by PCR using the Advantage2 kit (Clonetech) to amplify 400–600 bp fragments of the open reading frame from cDNAs generated by the SuperScriptIII kit (Invitrogen). These fragments were scanned for variations by comparing obtained sequence from mutants and siblings to sequences deposited in Ensembl (http://uswest.ensembl.org/Danio_rerio/Info/Index). To pinpoint the *tm246a* lesion, primers were designed to amplify the genomic DNA around the splice acceptor site of exon 9 (forward primer, TCGTAAAAGCTGCAGACCCTA; reverse primer, TGATCAGACAGCTGGTGGAA). Before sequencing, all PCR products were purified using the QIAquick Gel Extraction kit (Qiagen).

### Microscopy

DIC and *in situ* images were captured with a Leica DMLB widefield microscope equipped with an AxioCam MRm (for DIC) or an AxioCam MRc 5 (for *in situ*) camera (Zeiss) using AxioVision acquisition software (Release 4.5, Zeiss). All other images were captured on a Zeiss LSM 700 upright confocal microscope using the Zen acquisition software (2009 release, Zeiss). To view live larvae or the lateral cristae of fixed larvae, animals were mounted in 1% low melt agarose (Life Technologies) dissolved in E3 embryo medium and imaged with a 63x/0,95 water immersion lens. To view superficial neuromasts after immuno-labeling, larvae were mounted in Elvanol (0.1 M Tris pH 9.0, 10% polyvinyl alcohol, 88–89% hydrolyzed, 30% glycerol and 1% DABCO) and imaged with a 63x/1,4 oil lens.

### Vital Dyes

For the FM 1-43 experiments, zebrafish larvae were incubated for 20 sec in E3 containing 3 uM N-(3-Triethylammoniumpropyl)-4-(4-(Dibutylamino)styryl)Pyridinium Dibromide (FM 1-43, Life Technologies). For the Sodium Green experiments, zebrafish larvae were incubated for 20 min in E3 containing 10 uM tetra(tetramethylammonium) salt (Sodium Green, Molecular Probes) and 1% DMSO at room temperature in the dark. After either treatment, larvae were washed with E3 plus 0.02% 3-amino benoic acid ethylester (MESAB, Western Chemical Inc.) for 1–2 min at room temperature.

### Immunofluorescence

Zebrafish larvae were fixed in 4% paraformaldehyde in PBS for 4.5 hrs or overnight depending on the age of the larvae and the primary antibody used. Primary and secondary antibodies were incubated overnight at 4°C. The mouse IgG_1_ NKA antibody was obtained from the Developmental Studies Hybridoma Bank. Primary antibody was diluted (1∶500) in 1% BSA, 0.5% fish skin gelatin, 0.02% sodium azide in 1×PBS plus 2% goat serum. After primary antibody incubation, larvae were washed in PBS with 0.01% Tween 6 times over 3 hrs and then incubated in anti-mouse-Alexa 488, or anti-mouse-Alexa 647 (Life Technologies) (1∶1000) overnight. To label actin, phalloidin conjugated to Alexa 488 (Life Technologies) was added at 1∶500 alone or during incubation with secondary antibodies.

### Image Analysis

Images were processed using ImageJ software. Maximal z-projections of confocal images were generated to quantify the amount of NKA in the lateral crista. Integrated intensity of the z-projections was measured in MetaMorph software (Molecular Devices). To quantify colocalization of NKA and phalloidin labeled actin in the stereocilia, sections every 3 µm were analyzed from confocal stacks of lateral cristae. Regions encompassing stereocilia were defined using the phalloidin stain as a guide and included the base of the bundle just above the cuticular plate. Colocalization was performed using MetaMorph; percent of overlap is defined as the percent of phalloidin positive pixels that overlap with NKA positive pixels.

Profiles of NKA fluorescence were plotted using ImageJ. At least three HCs from lateral cristae from two experiments were used for analysis. The regions to measure profiles were drawn across cells from single sections of a z series. The regions were 1.0 µm thick and wide enough to cross both membranes of the cell. The regions were placed equidistant from the cuticular plate and the nucleus. Membrane NKA was calculated as the average of grey values 1.0 µm wide about the peak of NKA labeling at the edge of the cell. Cytoplasmic NKA was quantified using a 1.0 µm wide centrally located area between these regions.

To quantify length and width of stereociliary bundles, maximal z-projections of individual bundles from confocal images were made and measurements were made in ImageJ using the line tool. To quantify Sodium Green fluorescence, a circular region 6 µm in diameter was drawn around the base of the cell with the circle tool at a plane in the center of the nucleus of each cell. The DIC channel was used to determine the center of the nucleus for each cell analyzed.

### *In Situ* Hybridization

Phenylthiourea (PTU) treated larvae were fixed overnight in 4% paraformaldehyde, washed in PBS +0.1% Tween and stored in methanol at −20°C. The probe template used to detect *ap1b1* transcript corresponds to 1772–2114 bp of the coding region. The *in situs* were performed according to an established protocol [23]. The GenBank accession number for *ap1b1* mRNA is NM_001128530.1.

### Electron Microscopy

Whole larvae (5 dpf; n ≥5) were anesthetized with 0.02% MESAB and then fixed by immersion in 2.0% glutaraldehyde and 1.0% paraformaldehyde in normal solution (145 mM NaCl, 3 mM KCl, 1.8 mM CaCl_2_, 10 mM HEPES, pH 7.2) overnight to several days at 4°C. Specimens were fixed with 1.0% OsO_4_ in H_2_O for 10 min on ice, followed by fixation and contrast with 1.0% uranyl acetate for 1 h on ice, and then dehydrated with several steps in ethanol and embedded in Epon. Ultrathin sections were stained with lead citrate and uranyl acetate.

### Calcium Imaging and Hair Bundle Stimulation

Calcium imaging and analysis was performed as described elsewhere [19].

To deflect hair bundles, we used a fluid-jet composed of a pressure clamp HSPC-1 (ALA Scientific, New York) attached to a glass micropipette (tip diameter 25–35 µm for 2 dpf, and 40–50 µm for 3 and 5 dpf), positioned 100 µm from a given neuromasts. Deflections were sustained for the duration of the stimulus, and confirmed visually. A 25 mmHg fluid-jet stimulus was used to deflect hair bundles approximately 5–10°.

## Results

### Positional Cloning of *skylab* and Expression of *ap1b1*

**Figure 1:**
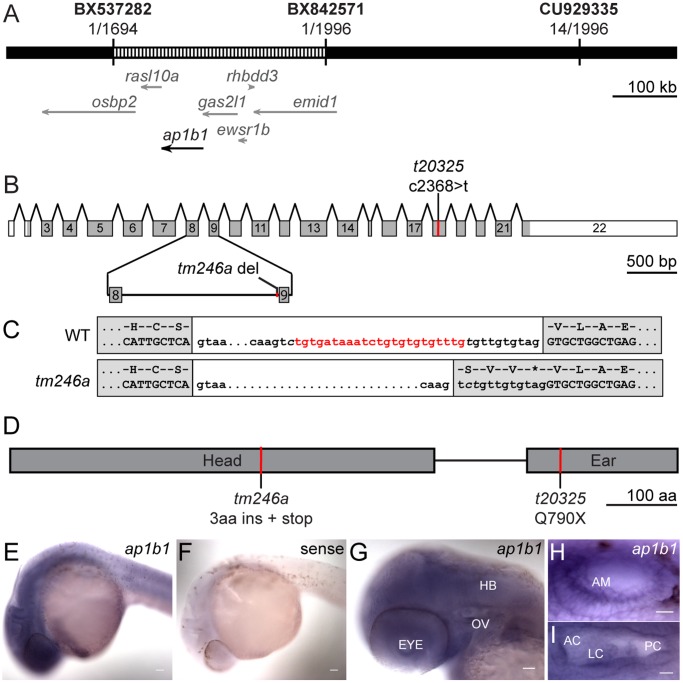
Positional cloning of *skylab* mutations and expression of *ap1b1*. ***A,*** A diagram of the 330 kb *skylab* critical interval (striped region) obtained through mapping of the *tm246a* allele. The critical interval encompasses the coding regions of five annotated genes as well as part of two other genes. ***B*** **,** An exon diagram of the *ap1b1* gene. The coding region is depicted in grey and the 5′ and 3′ UTRs are depicted in white. The locations of the *t20325* C-T transition the *tm246a* 23 bp deletion between exons 8 and 9 are indicated. ***C*** **,** The nucleotides deleted from the splice acceptor site between exons 8 and 9 in the *tm246a* mutant are highlighted in red in the WT transcript. The resulting translations are shown above the WT and *tm246a* transcripts. ***D*** **,** Diagram showing the location of *tm246a* and *t20325* mutations in the Ap1b1 protein. ***E,*** *ap1b1* is expressed ubiquitously at 24 hpf. ***F,*** Sense control for *ap1b1 in situ* experiments. ***G,*** Expression of *ap1b1* persists in the head at 48 hpf. Scale bars in E-G, 5 µm. ***H, I,*** Magnified images of the developing ear at 24 and 48 hpf, respectively. Scale bars in H and I, 10 µm. HB, hindbrain; OV, otic vesicle; AM, anterior macula; AC, anterior crista; LC lateral crista; PC, posterior crista.

In two independent forward genetic screens for mutants that fail to respond normally to an acoustic tap, two recessive alleles of *skylab* were isolated, *tm246a* and *t20325* [18] (Tübingen 2000 Screen Consortium). Both mutants develop normally, but do not inflate their swimbladders and die around 8–9 days post-fertilization (dpf). To identify the mutations in *skylab*, we undertook a positional cloning approach. The *tm246a* lesion was finely mapped to a 330 kb critical interval on chromosome 5 using approximately 1900 homozygous larvae. This critical region contained no gaps and included all or part of 7 genes (Figure 1A). Amplification and sequencing of cDNAs from these 7 genes subsequently revealed that both alleles have early stop mutations in the *ap1b1* gene, which encodes the β-subunit of the AP-1 complex.

The *tm246a* mutation arises from a 23-base pair deletion near the splice acceptor site in intron 8 (Figure 1B and C). Although the core AG sequence of the acceptor site is intact, the deletion causes mis-splicing and results in an in-frame inclusion of four codons into the coding region between exons 8 and 9. The added fourth codon is a stop codon (Figure 1C and D). This mutation truncates the predicted Ap1b1 protein in the middle of the head domain, which is required for cargo binding and complex assembly, and completely removes the ear domain, which binds to clathrin [24]. The *t20325* allele contains a single c2340>t mutation changing a Glu residue in the ear domain to a stop in the open reading frame (Figure 1B and D). This mutation may disrupt clathrin binding and therefore formation of the clathrin lattice. The two early stop mutations uncovered in the *tm246a* and *t20325* alleles provide strong evidence that the phenotype observed in *skylab* mutant larvae is due to mutations in *ap1b1*.

To determine where *ap1b1* is expressed in developing zebrafish, we used *in situ* hybridization. In other species, the AP-1 complex is expressed in all cell types. We observed that *ap1b1* is expressed throughout the embryo at 24 and 48 hours post-fertilization (hpf; Figure 1E–I), including in the developing ear at both stages. The ubiquitous expression of *ap1b1* is consistent with published expression data of the AP-1 µ-subunits [25]. These results also support a role for *ap1b1* in the sensory epithelium of the auditory and vestibular system.

### *ap1b1* Mutants Exhibit Auditory and Vestibular Defects

**Figure 2:**
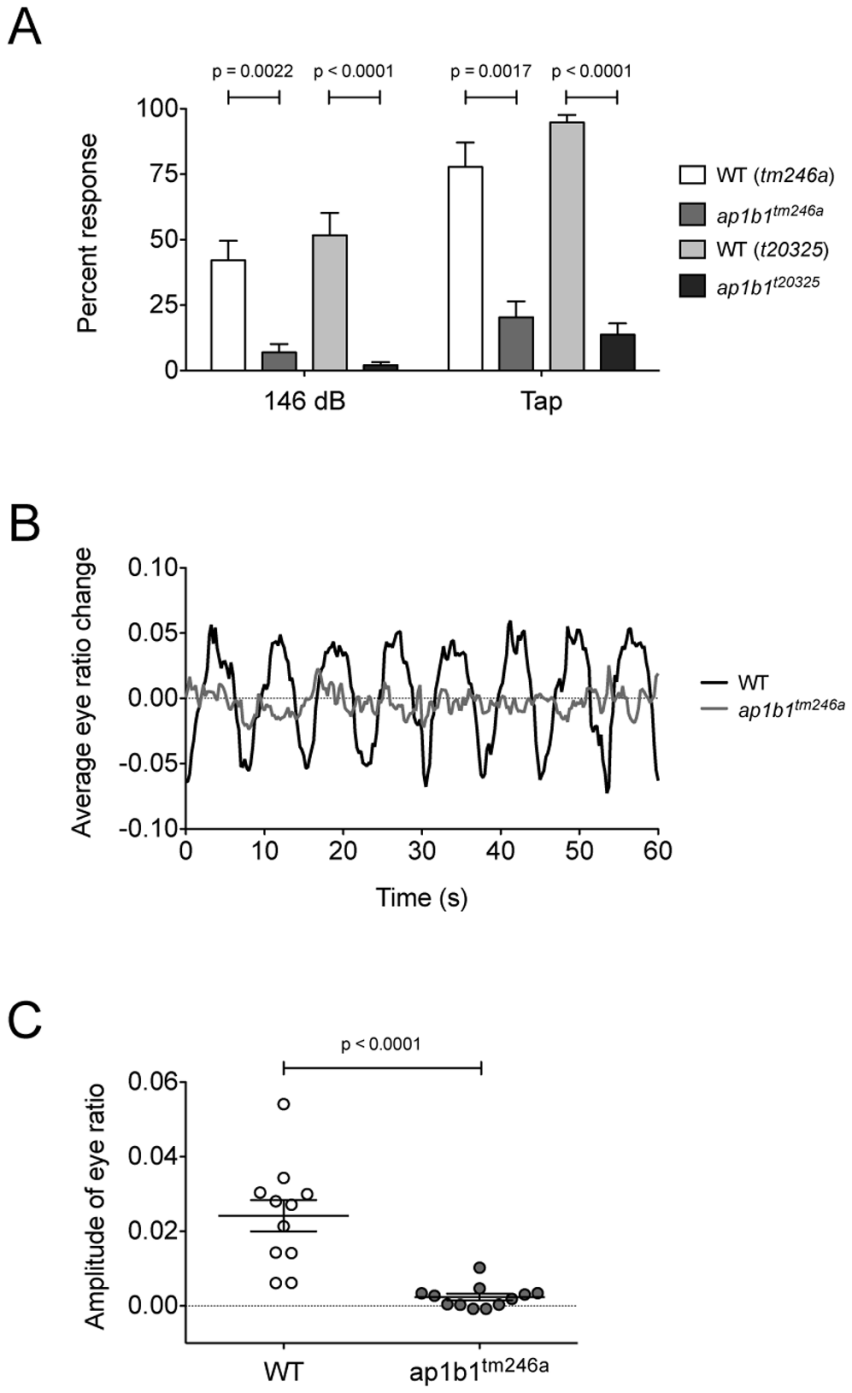
*ap1b1* mutants have deficits in auditory and vestibular behavioral responses. ***A,*** Graph showing the average startle response to either a 1000Hz stimulus at 146 dB or a tap stimulus of both mutants (*tm246a*: n = 9, *t20325*: n = 23) and their WT siblings (*tm246a*: n = 15, *t20325*: n = 19). ***B,*** Averaged traces of vestibular-induced eye movements from WT siblings (n = 11) and *tm246a* mutants (n = 12) over 60 sec. ***C,*** Average of peak amplitude of vestibular-induced eye movements at 0.25 Hz. Each dot represents one eye from an individual larva at 5 dpf (WT: n = *11, tm246a:* n = 12). A Mann-Whitney U-test was used to compare differences between mutants and WT siblings.

HCs populate two sensory organs, the inner ear and lateral-line organ, in aquatic vertebrates such as fish and frogs. In the inner ear, HCs are organized into several epithelial patches called cristae or maculae and mediate auditory and vestibular responses. In the larval lateral-line organ, HCs form superficial clusters called neuromasts. Neuromasts are responsible for sensing water movements and are important for schooling and predator/prey behaviors. At the free-swimming stage of development, 4–5 dpf, the zebrafish inner ear is fully functional and larvae maintain an upright position while resting and exhibit a robust startle reflex to acoustic/vibrational stimuli. Qualitative characterization of the *tm246a* allele revealed that mutant larvae are only partially sensitive to an acoustic tap stimulus [26]. We quantified the acoustic startle reflex at 5 dpf and observed that both mutant alleles have a significantly reduced startle reflex compared to their WT siblings in response to either a loud pure-tone stimulus (146 dB, 1000 Hz), or a multispectral tap stimulus (Figure 2A).

In accordance with having an auditory deficit, *ap1b1* mutants also swim in a circular pattern and fail to maintain an upright resting position, indicating that they also have balance defects. To quantify the deficit in vestibular function, we tested vestibular-induced eye movements in *tm246a* mutant and sibling larvae at 5 dpf [21]. Upon head rotation, mutant larvae moved their eyes in response to visual cues (data not shown), suggesting that their vision is not disrupted. In contrast to WT siblings, when rotated in the dark, vestibular-induced eye movements were nearly undetectable in *tm246a* mutant larvae (Figure 2B). A similar response was also observed in *t20325* mutants (data not shown). The average amplitude of the vestibular-induced eye movement in *tm246a* mutants was severely reduced compared to WT siblings (Figure 2C). Combined, these results demonstrate that *ap1b1* mutants have pronounced deficits in both auditory and vestibular function.

### Mechanotransduction is Disrupted in ap1b1 Mutant HCs

**Figure 3:**
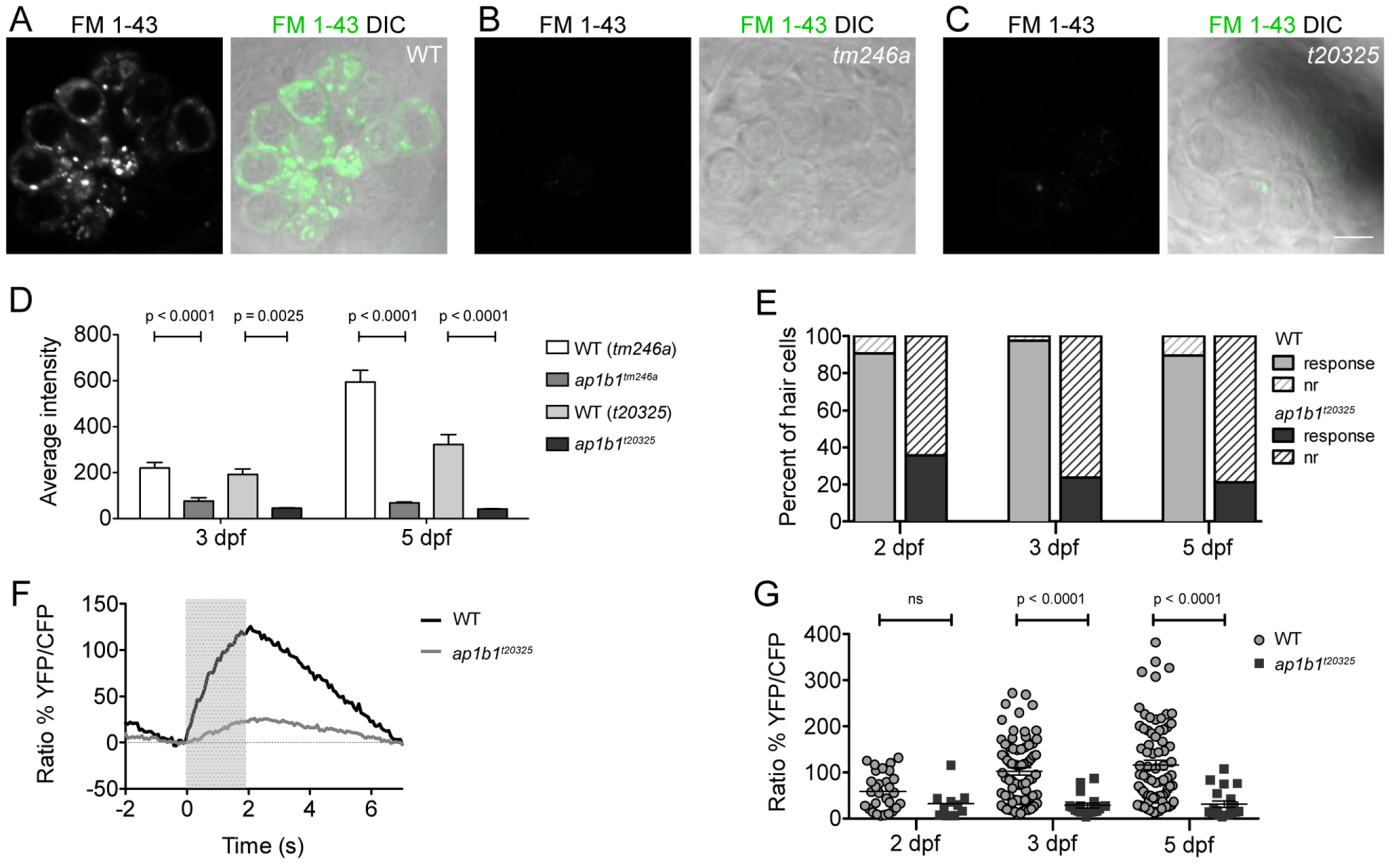
*ap1b1* mutants have deficits in HC mechanotransduction. ***A–C,*** FM 1-43 label of neuromast HCs in WT, *tm246a* and *t20325* mutants at 5 dpf. Scale bars, 5 µm. ***D,*** Average intensity (A.U.) of FM 1-43 label in *tm246a* and *t20325* mutants quantified at 3 dpf (*tm246a*: WT n = 18, mutant n = 5; *t20325*: WT n = 16, mutant n = 8 neuromasts) and 5 dpf (*tm246a*: WT n = 9, mutant n = 10; *t20325*: WT n = 12, mutant n = 11 neuromasts) from at least 3 larvae along with WT, age-matched siblings. ***E,*** The proportion of HCs displaying calcium transients in response to a water-jet stimulus (solid) compared to those that do not respond (nr = non-responders, hatched lines). The percent of non-responding HCs in the *t20325* mutants is greater than the percent non-responders in WT at all stages of development assayed; Chi Squared test, p<0.0001. ***F,*** Trace representing the average calcium responses to a 2 sec water-jet stimulus from 5 dpf WT and *t20325* mutant larvae (n = 20 HCs). The grey box indicates the timing of the water-jet stimulus. ***G,*** Dot plot showing calcium transients in WT and *t20325* larvae at 2, 3 and 5 dpf (non-responders were excluded). Each point represents an individual HC. Error bars represent SEM and statistical analysis was performed using a Mann-Whitney U-test.

Behavioral deficits in hearing and balance may be due to defects in either the peripheral or central components of the auditory/vestibular system. A previous study of *tm246a* larvae noted a reduction in FM 1-43 label of HCs and reduced HC sensitivity to ototoxic drugs [26], [27]. As both FM1-43 and ototoxic drugs are thought to permeate HCs with functional transduction channels, FM 1-43 labeling and drug sensitivity are commonly used as indicators of HC function [28], [29], [30], [31], [32]. The behavioral deficits reported here and the previous studies with *tm246a* larvae suggest that mechanotransduction is only partially functional in *ap1b1* mutants. To determine the onset of hair-cell dysfunction, we measured the intensity of FM 1-43 label in mutant lateral-line HCs at 3 dpf, when many HCs are still maturing, and at 5 dpf, when the majority of the HCs are mature. We observed a striking reduction in the amount of FM 1-43 label in *ap1b1* mutant HCs compared to WT at 5 dpf (Figure 3A–C). The reduction was comparable in both mutant alleles and was seen as early as 3 dpf (Figure 3D). At 5 dpf, when the proportion of mature HCs is greater than at 3 dpf, the difference between mutants and WT siblings in FM 1-43 label was more prominent. The pronounced reduction in FM 1-43 label over time suggests that both mutant alleles have the same effect on mechanotransduction.

As FM 1-43 labeling of lateral-line HCs is robust only at later stages of development [19], we sought to determine if mutations in *ap1b1* also had an effect on mechanotransduction at early stages, when hair cells first become mechanically sensitive. To assay early hair-cell activity, we used the transgenic line *Tg(myo6b:D3cpv)* that stably expresses D3-cameleon in HCs and examined evoked calcium transients, which is a more sensitive method for measuring HC function [19]. D3-cameleon is a genetically encoded calcium indicator that uses fluorescence resonance energy transfer (FRET)-based technology to measure changes in intracellular calcium [19], [33]. For our experiments, we mechanically stimulated lateral-line HCs with a fluid jet and recorded evoked calcium transients in individual HCs at early stages of development (2 dpf), earlier than when FM 1-43 labeling is detectable [19]. We crossed the *Tg(myo6b:D3cpv)* line into the *t20325* background, and compared the responses in WT and mutant HCs. In *t20325* fish, far fewer HCs responded to the stimulus than in WT siblings at all stages examined (Figure 3E). Of the *t20325* mutant HCs that responded to stimuli, the responses were comparable to those in WT at 2 dpf, but thereafter were significantly reduced compared to the WT siblings (Figure 3F and G). The decrease in HC activity that we observe in *ap1b1* mutants is consistent with the reduction of acoustically-evoked hindbrain calcium transients reported in an earlier study of this mutant [26]. The calcium imaging experiments demonstrate that at all stages of development, the majority of HCs fail to respond to mechanical stimuli. Over time, the calcium responses in mutant HCs remain low and fail to increase in size as seen in WT cells (Figure 3G). Together, the FM 1-43 and calcium imaging data indicate that mechanotransduction in *ap1b1* mutants is compromised at an early stage in HC development.

**Figure 4:**
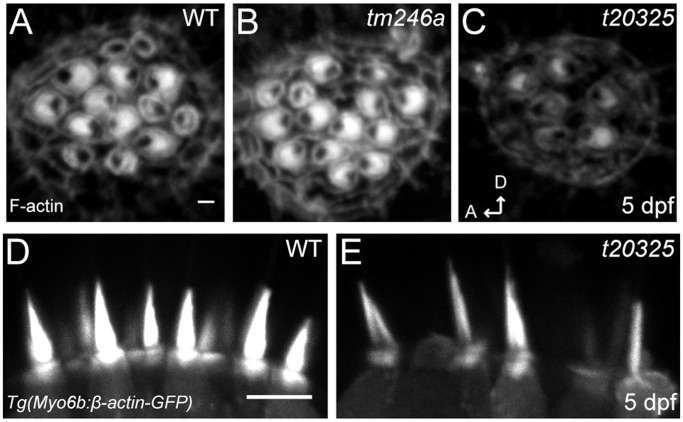
Stereociliary bundles of *ap1b1* mutant. ***A–C,*** Representative confocal images of neuromast hair bundles in WT, *tm246a* and *t20325* mutants at 5 dpf. Bundles were viewed from a top-down angle and actin was labeled with phalloidin-Alexa 488. This view shows the planar cell polarity of hair-bundles. Scale bar, 1 µm. ***D, E,*** Side view of stereocilia from the lateral cristae of 5 dpf WT and *t20325* mutants in the *Tg(myo6b:βactin-GFP)* background (z-projections, 2 µm thick). Scale bar, 5 µm.

Aside from the lack of FM 1-43 labeling and reduced calcium transients, a common feature among zebrafish transduction mutants is splayed hair bundles [26]. Splaying can occur when components of the transduction complex are missing or mutated, notably the tip-link proteins Pcdh15 and Cdh23 [34], [35]. Because *ap1b1* mutants have reduced FM 1-43 labeling and calcium transients, we examined hair-bundle morphology by labeling actin filaments with fluorescently-tagged phalloidin. At 5dpf, the intensity of the label was reduced in *t20325* bundles (Figure 4A and C). Quantification of the amount of phalloidin labeling revealed a significant decrease in the amount of actin in mutant bundles when compared to WT bundles (WT: 61.5 A.U. ±5.1; *t20325:* 48.0 A.U. ±1.2, Mann-Whitney U-test: p = 0.0006). The *tm246a* mutants, however, showed no significant change in the amount of actin (Figure 4B, 60.5 A.U. ±5.1) compared to WT larvae. As suboptimal fixation can lead to mild splaying or morphological artifacts, we examined bundle morphology in live HCs using a transgenic fish that expresses GFP tagged β-actin in HCs, *Tg(m6b: β-actin-gfp)* [19]. In the *t20325* mutant background, we observed thinner hair bundles in the lateral cristae of mutants (Figure 4D and E). The width of the hair bundle at its base was significantly decreased (WT: 1.4 µm ±0.1; *t20325:* 1.2 µm ±0.1; Mann-Whitney U-test: p = 0.0002). The height of mutant bundles appeared to be unaffected compared to WT bundles (WT: 4.7 µm ±0.2, n = 80 bundles from 3 larvae; *t20325* mutant 4.6 µm ±0.2, n = 49 bundles from 3 larvae; Mann-Whitney U-test: p = 0.5885). Overall these data suggest that maintenance of the bundle structure is affected by the *t20325* mutation. The bundle defects are subtle, however, and are not likely to fully account for the strong reduction of FM1-43 labeling and calcium transients in *t20325* HCs.

### *Ap1b1* Mutants Show Degeneration of Inner Ear and Lateral-line Neuroepithelia

**Figure 5:**
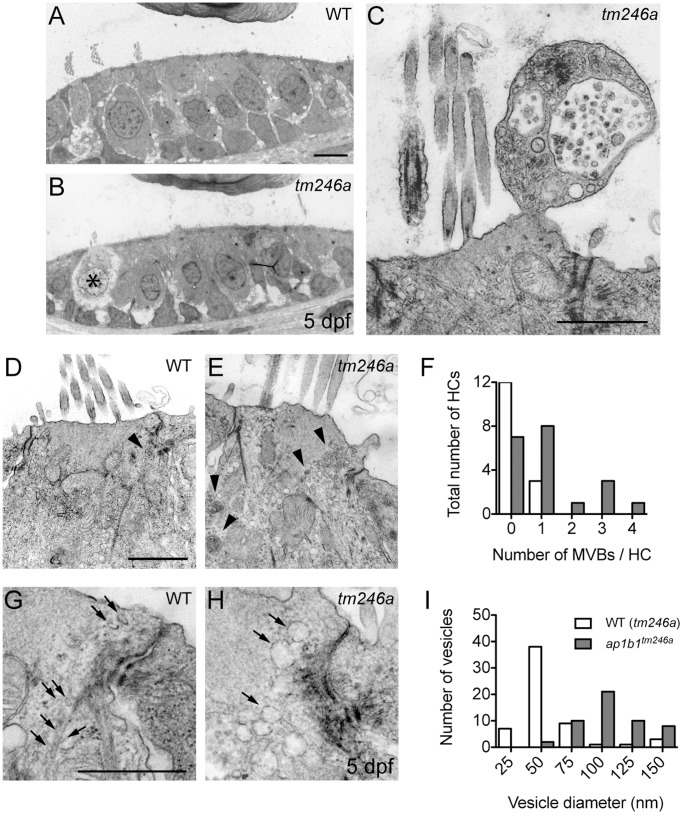
Decreased cell integrity and an increased number of intracellular membrane compartments in *ap1b1* mutant HCs. ***A, B,*** Comparable sections of the anterior macula of WT and *tm246a* mutant at 5 dpf. An asterisk indicates a breakdown of cytoplasm in a *tm246a* mutant HC. Scale bars, 3 µm. ***C,*** Example of an apical bleb extruding from a mutant HC containing several vesicular compartments and a multivesicular body. Scale bar, 1 µm. ***D–E,*** Comparable close-ups of WT and *tm246a* mutant HCs just below the cuticular plate. Scale bar, 1 µm. In *tm246a* mutant HCs, more multivesicular bodies (arrowheads) were present compared to WT HCs. ***F,*** Quantification of the observed number of multivesicular bodies in WT sibling and *tm246a* mutant HCs. ***G–H,*** Sections of HCs near the tight junctions showing an increased number of large vesicles in the mutants compared to WT. Vesicles in WT are indicated with arrows. ***I,*** Observed sizes of vesicles in WT sibling and *tm246a* mutant HCs. For quantification in ***F*** and ***I***, WT: n = 15, *tm246a*: n = 20 HCs.

In previous work, larvae carrying the *tm246a* allele were classified as a HC degeneration mutant due to the cellular defects, such as blebbing, in the inner ear sensory epithelia detectable with light microscopy [26]. Given the signs of degeneration and the role of the AP1 complex in protein sorting, we examined the cellular morphology and membrane compartments within hair cells in more detail using TEM (Figure 5).

In sections of the anterior macula of the ear (5 dpf), interstitial edema was evident in mutant sensory epithelia, with many extracellular spaces present between cells (Figure 5A and B). We also observed that the overall health of HCs was compromised in *tm246a* mutants (Figure 5B). Occasionally, we observed HCs largely devoid of cytoplasm in *tm246a* mutants, indicative of an abnormal physiological state (Figure 5B, asterisk). Another indication of an abnormal physiological state was the increase in multivesicular bodies in mutant HCs compared to WT (Figure 5D and E, arrowheads, quantified in Figure 5F). In addition to an increased number of multivesicular bodies, the number and size of membranous compartments localized below the cuticular plate were greater in mutants compared to WT HCs (Figure 5G and H, arrows, quantified in Figure 5I). Both enlarged vesicles and multivesicular bodies were not restricted to the cell body but could also be seen populating the blebs being extruded apically, adjacent to the bundle of stereocilia (Figure 5C). These observations of interstitial edema and blebbing suggest that mutations in *ap1b1* negatively affect ionic homeostasis in HCs, and the integrity and maintenance of HC membrane compartments.

### Localization of the Basolateral Membrane Protein NKA in *ap1b1* Mutants

**Figure 6:**
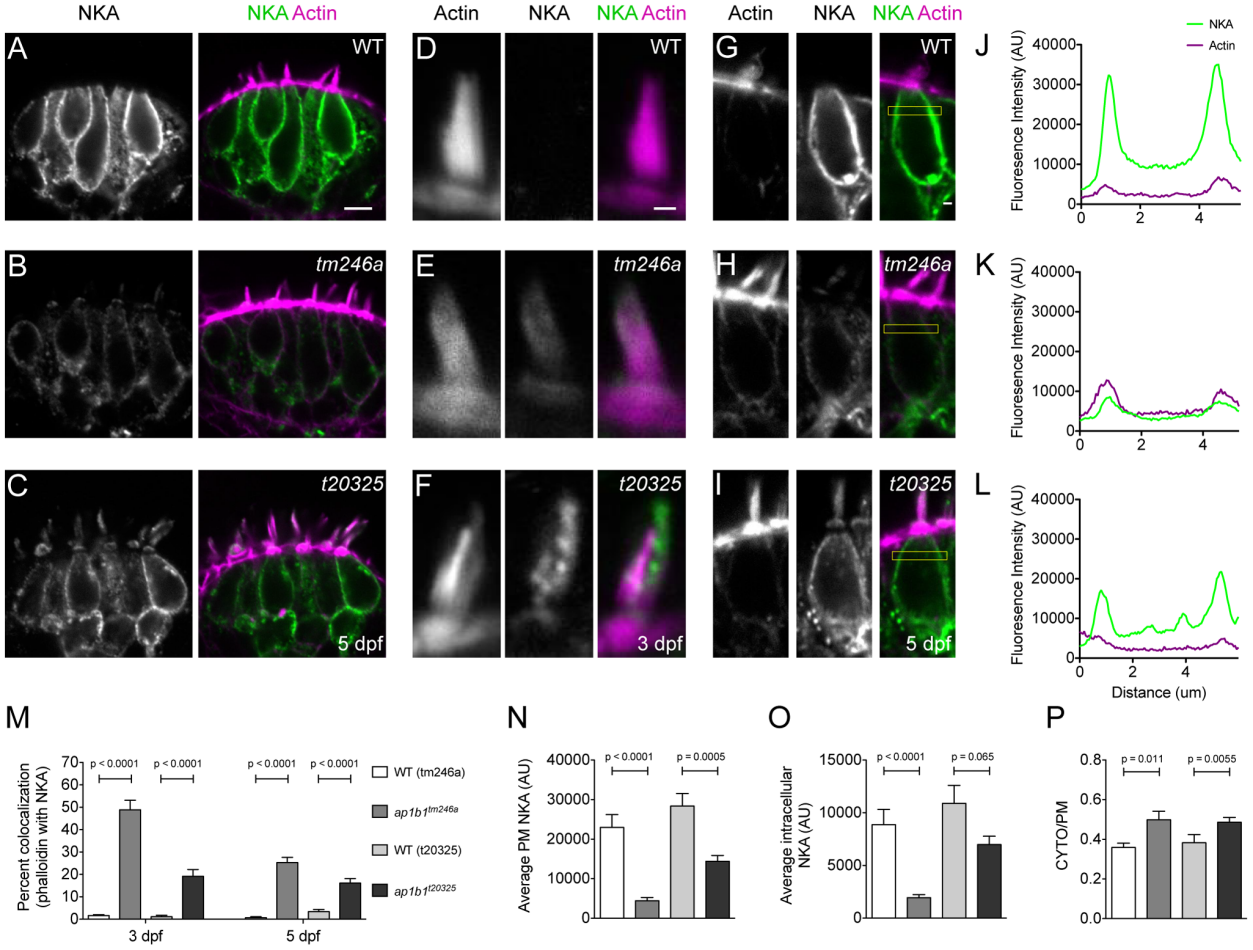
NKA is missorted to hair bundles in *ap1b1* mutant HCs. ***A–C,*** NKA antibody label of the lateral crista of WT and *tm246a* and *t20325* mutants at 5 dpf, respectively. Scale bar, 5 µm. ***D–F,*** Magnified examples of a single representative hair bundle in the cristae in WT, *tm246a* and *t20325* mutants at 3 dpf. Scale bar, 1 µm. ***G–I,*** Representative HCs from which fluorescence profile plots were obtained. Yellow boxes indicate the region used for generating profile plots. Scale bar, 1 µm. ***J–L,*** Profile plots showing fluorescence intensity of the distribution of NKA immunolabel in WT and *tm246a* and *t20325* mutant HCs shown in ***G, H*** and ***I,*** respectively. The green trace indicates NKA immunolabel and the magenta trace indicates phalloidin labeling. ***M,*** Quantification showing the average percent of NKA positive phalloidin pixels in WT and mutant stereocilia at both 3 (*tm246a*: WT n = 45, mutant n = 37; *t20325*: WT n = 39, mutant n = 53 bundles) and 5 dpf (*tm246a*: WT n = 46, mutant n = 72; *t20325*: WT n = 44, mutant n = 62 bundles) from ≥4 larvae. ***N,*** Quantification of NKA fluorescence (A.U.) at the membrane at 5 dpf. ***N,*** Quantification of intracellular NKA fluorescence (A.U.). ***O,*** Quantification of intracellular NKA fluorescence (A.U.) at 5 dpf. ***P,*** The ratio of intracellular NKA (CYTO) to plasma membrane localized NKA (PM). For ***N–P,*** *tm246a*: WT n = 19, mutant n = 16; *t20325*: WT n = 18, mutant n = 21 HCs. Error bars in ***M–P*** represent SEM and statistical difference determined with a Mann-Whitney U-test.

Because the AP-1 complex has been implicated in transport of membrane proteins to the basolateral membrane, we hypothesized that the deficit in mechanotransduction and the cellular phenotypes in *ap1b1* mutants are due in part to defective localization of proteins within the HC plasma membrane. AP-1 cargo membrane proteins are sorted from the TGN or endosome to the plasma membrane through binding of the AP-1 µ or β-subunits to either tyrosine-based (YxxΦ) or di-leucine-based ([D/E]xxxL[L/I]) sorting signals, respectively [36], [37], [38], [39]. Sorting motifs are usually present within the cytoplasmic C-terminal tails of cargo membrane proteins. Despite the absence of a canonical sorting signal, previous evidence from cell culture experiments suggests that the α-subunit of the basolateral pump NKA is sorted by AP-1 [40]. We hypothesized that if NKA is an AP-1 dependent cargo, then the pump would be mislocalized in *ap1b1* mutant HCs. To examine AP-1 dependent sorting of NKA, we used an antibody that recognizes a highly conserved epitope found on all NKA α-subunits [41]. In WT animals at 3 and 5 dpf, this antibody labels the basolateral membranes of HCs of the lateral crista (Figure 6A) and lateral-line neuromasts (data not shown). NKA label is also observed on fibers that innervate the HCs and supporting cell membranes (Figure 6A–C), as well as other cell types such as ionocytes, neurons, and the pronephritic duct (data not shown). In the *tm246a* mutants, NKA was present in the basolateral membrane of HCs (Figure 6B), but the overall intensity appeared greatly reduced compared to WT (Figure 6A). In the *t20325* mutant, the overall intensity of NKA label appeared somewhat reduced compared to WT (Figure 6C), but not nearly to the same degree as the *tm246a* mutant. Strikingly, in larvae carrying either *ap1b1* mutant alleles, we observed that NKA was mislocalized to apical hair bundles (Figure 6E–F). In contrast, we never observed immunoabeling of NKA in WT hair bundles (Figure 6D). Quantification of NKA colocalization with phalloidin-labeled bundles at 3 and 5 dpf showed that a significant amount of NKA was missorted to the hair bundle at both developmental stages (Figure 6M). These data indicate that without a functional AP-1 complex, NKA is sorted indiscriminately to both basal and apical compartments in HCs.

To determine how efficiently NKA is targeted to the plasma membrane, we plotted the fluorescence profile of NKA across individual HCs and quantified the amount of NKA at the plasma membrane (Figure 6G–L). Both mutant alleles showed reduced NKA expression at the HC plasma membrane (Figure 6N). Given that NKA is reduced at the plasma membrane, we attempted to address whether NKA had accumulated within the cell body of HCs by quantifying intracellularly localized NKA using the central region of the fluorescence profiles (Figure 6O). In addition to inefficient sorting of NKA to the plasma membrane, the amount of NKA localized to the intracellular compartment was also significantly reduced in the *tm246a* mutant allele compared to WT siblings. And though not significant, there was a trend towards reduction in *t20325* mutants. The reduction of both plasma membrane and intracellular levels of NKA suggests that in both mutants, NKA is degraded. The fluorescent profiles (Figure 6J–K) indicated that, despite an overall decrease in NKA levels, mutant HCs showed greater relative staining of NKA in the cytoplasm than WT HCs. We therefore calculated the ratio of intracellular to plasma membrane NKA signal and found that indeed the ratio (CYTO/PM) was significantly increased in HCs harboring either allele (Figure 6P). This result suggests that targeting of NKA to the plasma membrane is reduced in *ap1b1* mutant HCs.

### The Amount of Intracellular Na^+^ is Increased in *ap1b1* Mutant HCs

**Figure 7:**
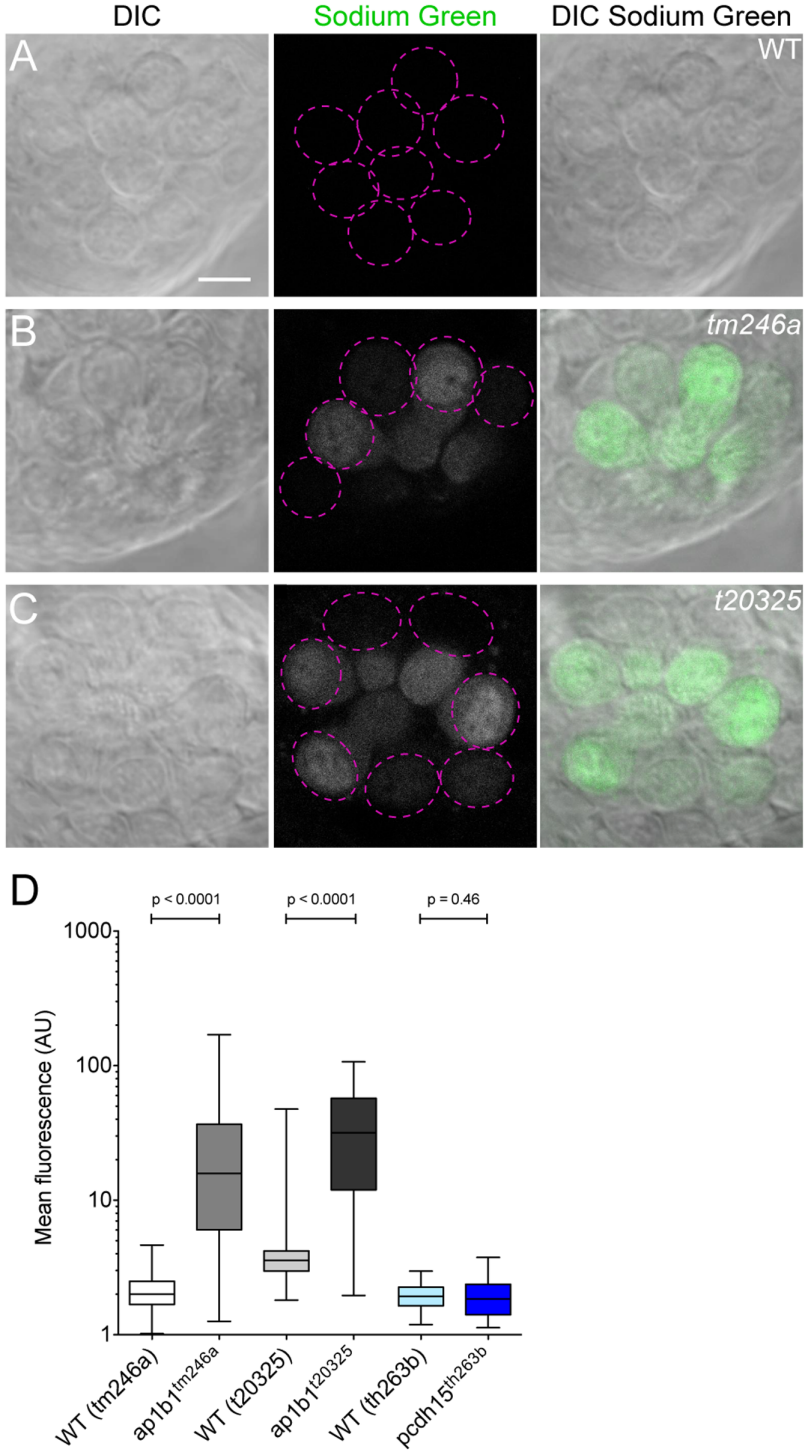
Increase of intracellular Na^+^ levels in mutant HCs. ***A–C,*** Sodium Green label in WT, *tm246a* and *t20325* mutant neuromasts. Dotted magenta circles outline HCs that were used for quantification in that plane of view. Scale bar, 5 µm. ***D,*** Quantification of Sodium Green label in *ap1b1* mutant, *pcdh15^th263b^,* and corresponding WT HCs. (*tm246a*: WT n = 124, mutant n = 90; *t20325*: WT n = 125, mutant n = 74; *th263b*: WT n = 100, mutant n = 84 HCs). Error bars represent SEM. Statistical analysis performed with a Mann-Whitney U-test.

In nearly all cell-types, NKA is the primary pump for maintaining the relatively low level of Na^+^ and high level of K^+^ inside the cell. With the observation that NKA is less abundant in the HC plasma membrane of both *ap1b1* mutants, we hypothesized that this reduction would lead to increased Na^+^ in HCs. This idea is consistent with previous reports that inhibition of NKA in goldfish HCs increases the concentration of intracellular sodium [42]. In addition, it has been demonstrated that blebbing in HCs can be triggered through an influx of Na^+^ [43], and blebbing occurs in both *ap1b1* mutants (Figure 5 and data not shown). To assay relative amounts of intracellular Na^+^, we incubated intact 5 dpf larvae in the fluorescent Na^+^ indicator Sodium Green. Compared to WT siblings, larvae carrying either mutant allele of *ap1b1* had significantly increased levels of Na^+^ in individual HCs (Figure 7A–C). Although the level of fluorescence varied among mutant HCs within a neuromast, overall it was increased in *ap1b1* mutant HCs compared to WT HCs (Figure 7D). This increase in Sodium Green label suggests that *ap1b1* mutants are unable to maintain appropriate intracellular Na^+^ levels. To determine whether an increase in Na^+^ levels was specifically due the trafficking defects in *ap1b1* mutants, and not secondary to its transduction defects, we tested another transduction mutant carrying the *pcdh15^th263b^* allele. At 5 dpf, *pcdh15* mutant HCs did not show an increase in intracellular Na^+^ (Figure 7D). This result suggests that Na^+^ build up is a unique consequence of mutations to *ap1b1*, and not due to the loss of mechanotransduction.

## Discussion

The polarized distribution of membrane proteins in epithelial cells is essential for cellular function and is accomplished in part through the AP-1 complex [44], [45]. It was previously thought that disrupting the entire AP-1 complex was lethal and hence, many of the seminal studies on this complex have been done in cell culture. Here we present to date the first reported mutations to the β-subunit of the AP-1 complex, which should disrupt all AP-1 dependent functions, and analyze the genetic and cellular consequences of this mutation in zebrafish. Our findings indicate that lesions in *ap1b1* disrupt the function and integrity of HCs in inner ear and lateral-line organs. Mutant HCs show progressive signs of degeneration, including blebbing and interstitial edema, and an accumulation of vesicles and multivesicular bodies. In addition, mechanotransduction is compromised, evidenced by the significant reduction in both FM 1-43 label and mechanically-evoked calcium transients. We propose that these defects are caused primarily by the missorting of basolaterally targeted proteins including NKA. Incorrect targeting of NKA leads to the presence of this pump in the apical hair bundle of mutant HCs. Loss of Ap1b1 function also results in a reduction of NKA at the basolateral membrane, leading to an increase in intracellular Na^+^. Collectively, these findings suggest that ion homeostasis and mechanotransduction are disrupted in HCs when basolateral proteins such as NKA are missorted to the apical surface.

### Auditory and Vestibular Functions are Especially Susceptible to *ap1b1* Mutations

Despite the ubiquitous expression of *ap1b1*, *ap1b1* mutants display no other obvious behavioral phenotypes aside from auditory and vestibular deficits. The development and function of cell types other than HCs appears to be unaffected in *ap1b1* mutants. This specific phenotype is unexpected considering the expression of *ap1b1* during development and the deleterious effects of lesions in the AP-1 complex in other species. For example, deletion of either the AP-1 γ or µ1A subunit in mice is embryonic lethal, as is removal of both AP-1 µ subunits in *C. elegans* [46], [47]. It is possible that the normal development of *ap1b1* mutants could be due to genetic redundancy, as paralogs are common in zebrafish. To date, however, we are unable to find a second copy of *ap1b1* in the latest assembly of the zebrafish genome (Zv9, July 2010 release). Alternatively, if Ap1b1 is required in other cell types, maternal mRNA may sustain embryos through earlier stages of development. Supporting the idea that the maternal contribution of mRNA ameliorates the loss of the AP-1 complex during early development, transcripts for both AP-1 µ subunits have been detected at the 2-cell stage [25].

Further corroboration that AP-1 complexes are critical for the function of the auditory and vestibular system comes from studies of AP-1 mutations in humans. Mutations in *AP1S1*, one of the three σ1 subunit genes in humans, results in MEDNIK (mental retardation, enteropathy, deafness, neuropathy, ichthyosis and keratodermia) syndrome [48]. This rare, recessive disorder causes congenital hearing loss, although the pathological consequences of loss of *AP1S1* function in the inner ear are not known. Knockdown of *ap1s1* in zebrafish results in the disruption of the integrity of embryonic keratinocytes and spinal cord development, however, knockdown was lethal at larval stages, precluding the assessment of auditory/vestibular function [48]. Nevertheless, the MEDNIK syndrome highlights the importance of AP-1 function in several epithelial and neuronal cell types.

### *ap1b1* is Required for Proper Localization of NKA in HCs

AP complexes selectively recognize cargo via intrinsic sorting signals, such as the di-leucine and tyrosine motifs [36], [37], [38], [39]. However, in the case of NKA, a canonical AP-1 sorting motif has not been identified in NKA. Instead, novel motifs within the α-subunit of NKA appear to be necessary for basolateral targeting in cell lines [40], [49], [50]. Additionally, unlike other well-known AP-1 dependent cargoes, the post-Golgi transport pathway taken by NKA to the plasma membrane does not involve recycling endosomes, but rather goes directly from the TGN to the plasma membrane [12]. In our study, we observe that a fraction of NKA is sent to the apical surface in *ap1b1* mutant HCs (Figure 6). As apical missorting of AP-1 dependent cargoes is a common outcome when AP-1 is disrupted [51], [52], our observations support the hypothesis that the AP-1 complex is required for sorting of NKA to the basolateral membrane.

In accordance with our observations that AP-1 is required for basolateral sorting of NKA, there is also a striking reduction in the level of NKA within the basolateral membrane in both *ap1b1* mutant alleles. Intracellular NKA was significantly reduced in the *tm246a* allele, and there was a trend towards reduction in the *t20325* allele. In contrast to NKA, we did not observe reductions in immunolabel of several components of the hair-cell synapse including the membrane α subunit of Cav1.3 (data not shown). A reduction in NKA protein level implies that the pump may be trafficked through a competing AP pathway, such as AP-3, which targets proteins to the lysosome, where NKA is likely degraded. Recent evidence in kidney cells demonstrates that AP-1 complexes are required for both stability and trafficking of the anion exchanger 1 (AE1) to the plasma membrane, suggesting that as observed with NKA in *ap1b1* mutants, mistargeted proteins are degraded as a consequence of missorting [53].

In addition to protein trafficking, the AP-1 complex is also important for maintaining the size and number of intracellular membrane compartments [54], [55]. Consistent with this role for AP-1, we noted changes within HCs, including the accumulation of multivesicular bodies and enlarged vesicles. It is not clear, however, if the differences in membrane compartments in *ap1b1* mutants are due to a block in AP-1-mediated protein trafficking, or to secondary, degenerative changes within the HCs. Indeed, dystrophic conditions in axons can lead to an increase in the number of multivesicular bodies, suggesting that formation of new multivesicular bodies is driven by pathological conditions [56]. Future work may address this distinction between direct and indirect consequences of AP-1 dysfunction.

### Missorting of NKA Causes a Na^+^ Imbalance in Mutant *ap1b1* HCs

In HCs, the activity of NKA is necessary to clear the build up of intracellular Na^+^ generated by other Na^+^-coupled transport activities [42]. Exchangers such as the Na^+^/H^+^ and Na^+^/Ca^2+^ pumps are thought to account for most of the Na^+^ flowing into HCs. Consistent with a role for regulating intracellular Na^+^, our data suggest that decreases in the cell-surface expression of NKA lead to an increase in intracellular Na^+^ concentrations in mutant HCs. Though overall Na^+^ levels are increased in mutant HCs, these levels were also highly variable among individual cells. The variability suggests that Na^+^ build-up may be progressive. We propose that the failure of NKA to balance Na^+^ loading within HCs leads to increased intracellular Na^+^. A rise in internal Na^+^ may disrupt several cellular processes, including Na^+^-coupled transport activity, and potentially the resting membrane potential. An increase in Na^+^ can also lead to overt signs of necrosis. A previous report demonstrated that excessive Na^+^ influx causes apical blebbing in cultured HCs [43]. Thus, elevated Na^+^ is likely to disrupt HC function and eventually lead to blebbing and cell death, which we observe in mutant HCs of both *ap1b1* alleles.

Polarized epithelial cells have the unique challenge of maintaining two functionally distinct domains of the plasma membrane. In polarized, electrically active cells, the expression and sorting of ion channels and transporters to distinct compartments is critical for cell activity. Based on our data, we propose that the AP-1 complex is vital for the correct sorting of NKA and the maintenance of ion homeostasis in HCs. As the lesions described here are the first reported mutations to the AP-1 β-subunit in a vertebrate animal model, the *ap1b1* mutant presents an opportunity for future studies to investigate how the β-subunit of AP-1 is involved in setting up and maintaining polarized distribution of proteins, a pathway important for many processes including development, cellular function and homeostasis.
